# Chandelier cells shine a light on the formation of GABAergic synapses

**DOI:** 10.1016/j.conb.2023.102697

**Published:** 2023-06

**Authors:** Benjamin Compans, Juan Burrone

**Affiliations:** Centre for Developmental Neurobiology and MRC Centre for Neurodevelopmental Disorders, King's College London, New Hunt's House, Guy's Hospital Campus, London, SE1 1UL, London, UK

## Abstract

Uncovering the wiring rules employed by neurons during development represents a formidable challenge with important repercussions for neurodevelopmental disorders. Chandelier cells (ChCs) are a singular GABAergic interneuron type, with a unique morphology, that have recently begun to shed light on the rules that drive the formation and plasticity of inhibitory synapses. This review will focus on the wealth of recent data charting the emergence of synapses formed by ChCs onto pyramidal cells, from the molecules involved to the plasticity of these connections during development.

## Introduction

**Figure 1:**
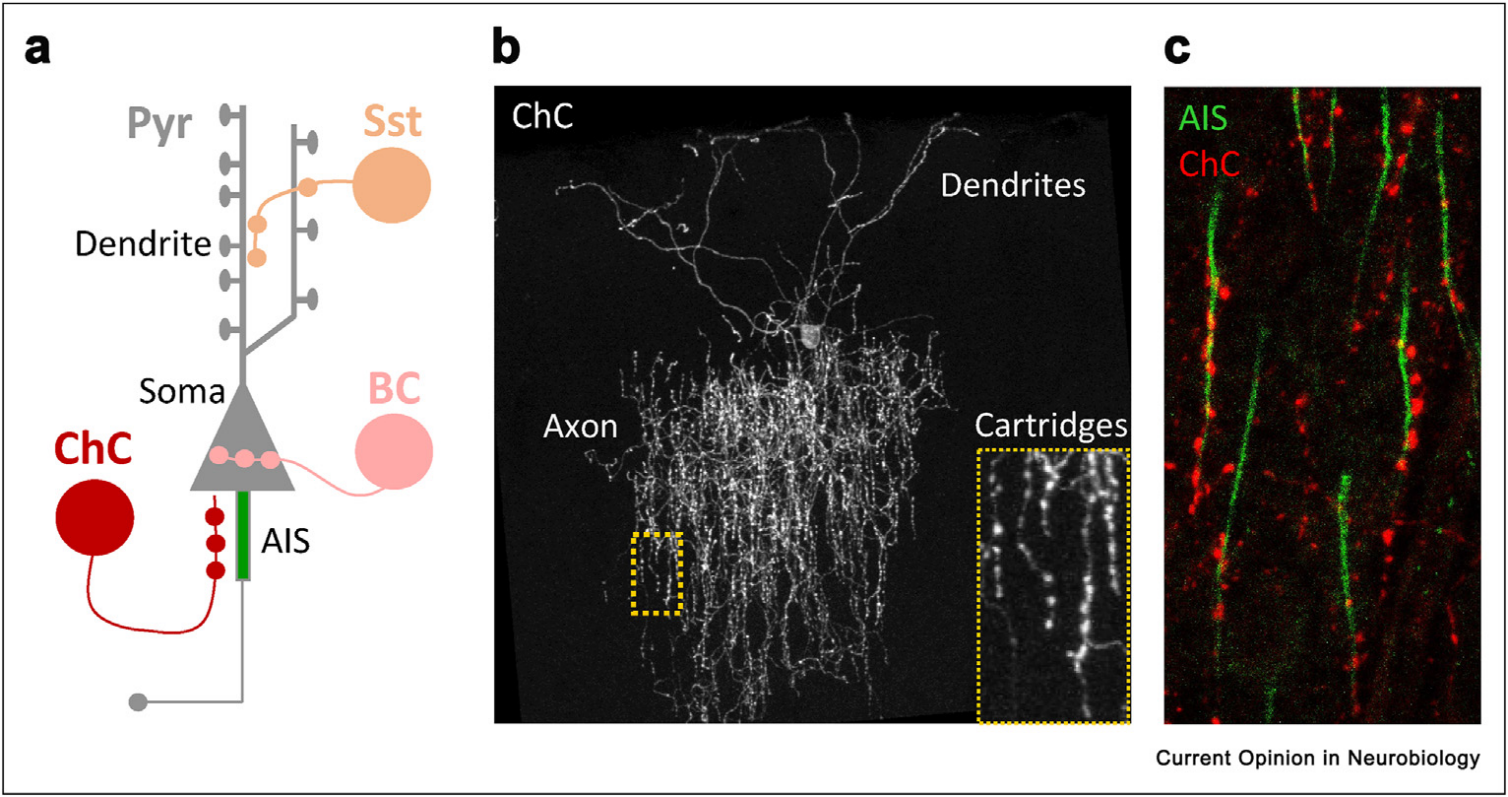
**Chandelier cells form axo-aoxnic synapses along the AIS. a**. Schematic representation of the 3 main subtypes of GABAergic interneurons and their connectivity onto a Pyramidal Neuron (Pyr, grey). Somatostatin expressing interneuron (SST, orange), Basket Cell (BC, pink) and Chandelier Cell (ChC, red) form synapses along the dendritic shaft, the soma and the axon initial segment of Pyramidal neurons, respectively. **b**. Confocal image of a Chandelier Cell from the L2/3 somatosensory cortex. Cartridges of ChC axo-axonic boutons are shown in the inset. **c**. Confocal image of ChC boutons (red) forming synapses along the axon initial segment of Pyramidal neurons (green).

The adult mammalian cortex is formed of essentially two main types of neurons: excitatory pyramidal neurons that release glutamate and inhibitory interneurons that release GABA and locally modulate pyramidal cell activity. Interneurons comprise a large and diverse population of GABAergic cells [1], with a subset forming direct contacts onto pyramidal cells, where they target-specific subcellular compartments (Figure 1a). The two major GABAergic cell types that directly innervate pyramidal cells are the somatostatin (SST)-expressing interneurons, which preferentially target dendrites, and parvalbumin (PV)-expressing interneurons. PV cells, in turn, can be further divided into two well-defined subclasses of interneurons: basket cells (BCs), which mainly target the soma, and chandelier cells (ChCs), which target the axon initial segments (AIS) of pyramidal neurons. The precise subcellular location of GABAergic synapses dictates the type of modulation they impart on pyramidal cells in adult brains, yet little is known about how this spatial arrangement emerges or what plasticity rules interneurons follow during development to fine-tune their connectivity. Indeed, the heterogeneity of interneuron subtypes has often been a stumbling block when trying to uncover their wiring rules. In this regard, genetic strategies that allow labelling of a specific interneuron subtype, early in development, of cells with well-defined morphological characteristics and a precise postsynaptic target would provide an ideal system to uncover the rules behind the formation, refinement, and plasticity of GABAergic synapses. Arguably, the newly developed transgenic mouse lines that label ChCs [2, 3, 4, 5] provide such a system and have therefore attracted much attention lately as a model cell for understanding the development and wiring logic of GABAergic interneurons in the brain. Here, we will focus on recent studies that have exploited these properties to study the emergence and plasticity of axo-axonic synapses.

## Chandelier cells: a unique interneuron in the brain

ChCs are fast-spiking, GABAergic interneurons found in many brain areas, including the cortex, hippocampus, and amygdala, and across different species, from rodents to humans [6, 7, 8, 9]. Although some morphological and molecular heterogeneity exists [10], ChCs have typically been considered a single interneuron class. Compared to other interneurons, ChCs are found only sparsely in the brain, a peculiarity that has delayed our understanding of the role they play in the brain. Their low numbers, however, are offset by their large, highly branched, and densely packed axon that contacts hundreds of neighbouring pyramidal neurons [11], suggesting an important role in controlling circuit function. A characteristic feature of these axons is that, in mature brains, they target the AIS of principal neurons, forming multiple axo-axonic synapses, known as cartridges, that appear as rows of vertically-aligned boutons along a postsynaptic AIS, resembling candlesticks on a chandelier [12] (Figure 1b–c). Since the AIS is the site of action potential initiation, ChCs are thought to tightly modulate the activity of neurons and circuits [13], although whether they are excitatory [5,14,15], inhibitory [16,17], or switch polarity depending on the state of network [18], remains an area of intense debate [19]. A more parsimonious explanation for some of these contradictory findings recently emerged from perforated-patch experiments showing that whereas GABA on dendrites switched from excitation to inhibition within the first postnatal week, GABA at the AIS remained depolarising for much longer, beyond the third postnatal week [20]. This delayed polarity switch, which was later confirmed with voltage imaging [21•], may account for some of the discrepancies in the field. However, these experiments, all of which have been carried out in brain slices, still remain to be tested *in vivo*. In fact, experiments assessing how interneurons as a whole modulate circuits early in development have only recently been carried out *in vivo*, with varying outcomes depending on brain region [22]. Similar experiments for single interneuron subtypes, such as ChCs, are still lacking. To date, the role of ChCs *in vivo* has only been explored in adult brains. Calcium imaging of ChC activity in the adult visual cortex (V1), for example, has shown that these cells do not respond to sensory stimuli [23•], even though electrophysiological recordings in the somatosensory cortex show they do receive sensory inputs [24]. Instead, their activity correlates well with measures of arousal state [25], such as pupil dilation [23•], and recent work has shown that ChCs mostly receive inputs from high-order cortical areas [17]. Importantly, *in vivo* activation of ChCs in the adult brain has so far mainly resulted in the inhibition of pyramidal cell activity [17,26], although not always [27].

In adults, specifically in layer 2/3 of the cortex, a single AIS will typically be contacted by multiple ChCs [11], ranging from 0 to 9 (3–4 on average), with each ChC contributing a single cartridge that forms anywhere from 1 to 9 boutons [11,13,21•,23•]. As a result, there is a large variability in the total number of axo-axonic boutons formed onto the AIS of different pyramidal cells, ranging anywhere from none to 30 boutons [23•]. Furthermore, the number of axo-axonic synapses varies quite dramatically across different brain areas, suggesting region-specific wiring rules [28]. Although it remains unclear what this heterogeneity means functionally nor how it arises in the first place, recent work in the cortex has shown that ChCs may actually target-specific pyramidal cell subtypes [17]. In the prelimbic cortex, for example, pyramidal cells projecting to the basolateral amygdala were preferentially innervated by ChCs when compared to contralateral projecting neurons [17]. In turn, ChCs received inputs mostly from local and contralateral projecting pyramidal neurons, but not those projecting to the BLA, suggesting that the innervation pattern of ChCs may be tightly orchestrated and tailored to specific microcircuits [17]. Understanding the emergence of the intricate connectivity between ChCs and their target cells remains an important area of research, particularly in the context of neurodevelopmental disorders [29]. Indeed, ChCs and specifically their axo-axonic synapses have been implicated in a number of disorders, including epilepsy [30, 31, 32] and schizophrenia [33,34].

## The development of ChCs and the formation of axo-axonic synapses

Compared to other interneurons in the brain, ChCs appear to do everything late. They are born late [3], show delayed migration into the cortex [3], undergo protracted developmental apoptosis [35•], and form synapses late [21•,^36^, 37•, 38]. Perhaps not surprisingly, they were also discovered late – it was only in the mid-1970s that these interneurons were first described [6,39,40], and shown to be the cells responsible for most of the axo-axonic synapses described in earlier EM work of the AIS [41, 42, 43]. As a result, we know relatively little about this elusive interneuron population. The advent of new transgenic mouse lines that label ChCs [2, 3, 4, 5] resulted in a wealth of new data that has begun to uncover their properties – from understanding their function *in vivo* to describing their development, particularly, the formation of their emblematic axo-axonic synapses.

**Figure 2:**
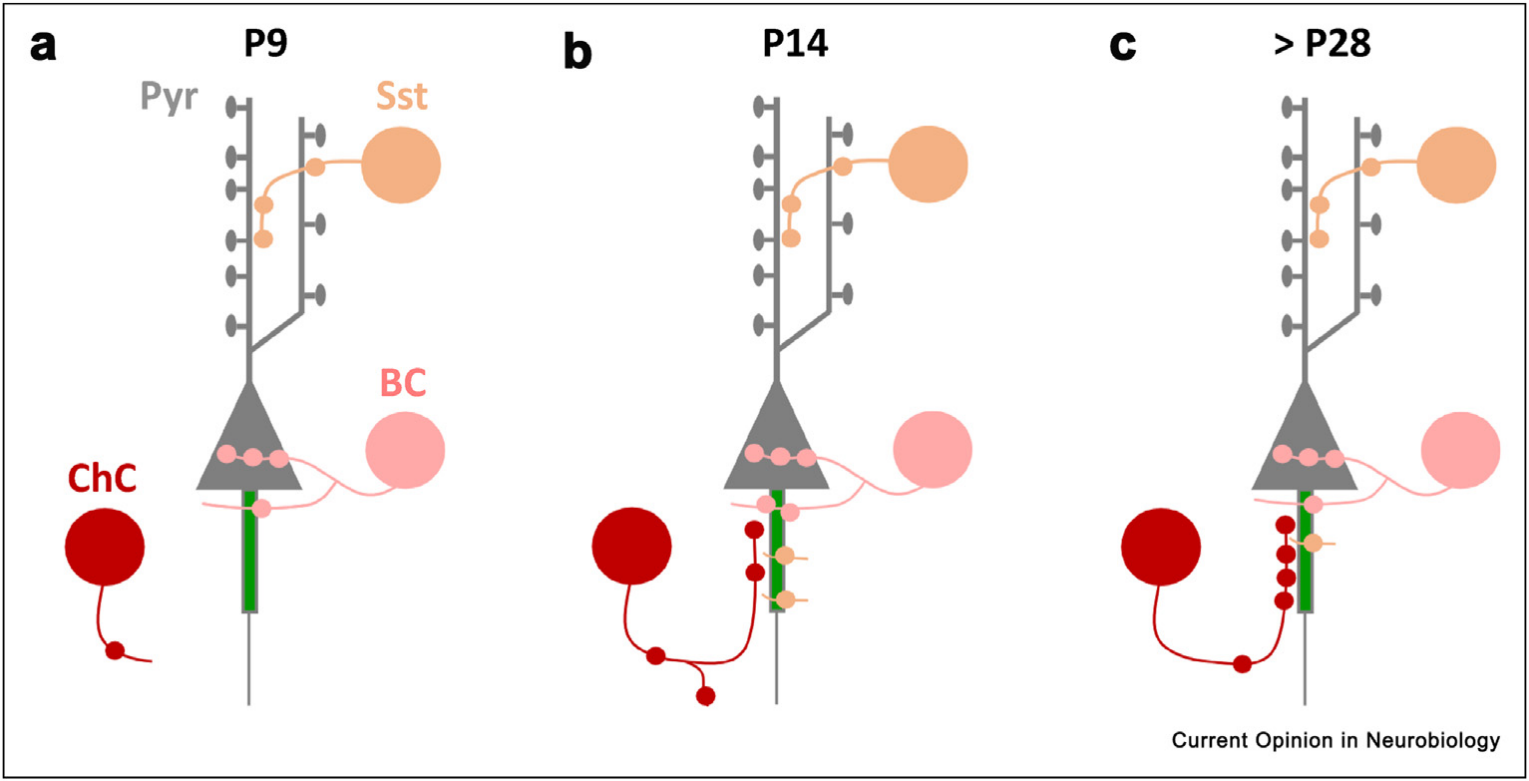
**Postnatal development of axo-axonic synapses.** Schematic representation of the synapses formed by Chandelier Cell (ChC, red), Basket Cell (BC, pink), and Somatostatin interneuron (SST, orange) onto Pyramidal neuron (Pyr) at P9 (**a**), P14 (**b**) and after P28. (**c**) During the first 2 postnatal weeks ChCs show an increase in both AIS-localised and off-target boutons. After the second postnatal week, ChCs rearrange their contacts by removing off-target synapses and specifically adding synapses at the AIS. In parallel, other inhibitory neurons that synapse preferentially onto other compartments (BC and/or SST), also form synapses at the AIS, particularly before P14, and are pruned away by P28.

ChCs are born in the ventral germinal zone (likely a remnant of the earlier medial ganglionic eminence) during the late embryonic stages (∼E17) and migrate to the cortex, reaching superficial layers by P2/P3 and deeper layers between P3 and P7 [3]. Although some axonal processes are present by P7, most ChCs show a strong period of axonal growth that, in the somatosensory cortex, typically occurs between P12 and P18 [21•]. In fact, individual ChCs can form mature axonal arbours within just a couple of days [21•], a narrow window that matches the formation of axo-axonic synapses along the AIS of pyramidal neurons [21•,^36^,37•]. This period of axonal development also results in changes in the connectivity pattern at the AIS, from innervation by a single ChC axon at P14, to poly-innervation (by around 4 ChCs) at P28 [38•], causing a dramatic increase in synapse number [38•]. A detailed time-course of synapse formation has shown that early pioneering ChC axons also form off-target contacts with pyramidal cells that are gradually eliminated throughout development [36], in agreement with EM studies showing that ChCs form multiple non-AIS contacts at P14 that are absent by P28 [38•]. To add to this highly dynamic period, axons from other interneuron subtypes (likely BCs) also contact the AIS, particularly early on in development. In fact, they represent more than two-thirds of all synapses along the AIS at P14. By P28, however, following the formation of ChC synapses and the pruning of non-ChC contacts, they dwindle down to around one-third of all AIS synapses, leaving ChCs as the main interneurons to innervate the AIS (Figure 2) [23•,38•]. Although the ongoing refinement of ChC synapses suggests pruning of incorrect synapses does take place, it is still unclear how ChCs achieve such a precise targeting of the AIS, a subcellular compartment that is only 30–40 μm long [44,45]. Whether chemotactic signals released locally at the AIS play a role in attracting axons directly to this region or whether contacts are made more broadly across pyramidal neurons and only kept at the AIS through specific retention molecules together with the removal of off-target contacts, is not yet known [38•]. However, recent efforts have begun to explore the molecular landscape that favours axo-axonic synapse formation and retention at the AIS in the hope of better understanding these events.

## Illuminating the molecular landscape of axo-axonic synapses at the AIS

**Figure 3:**
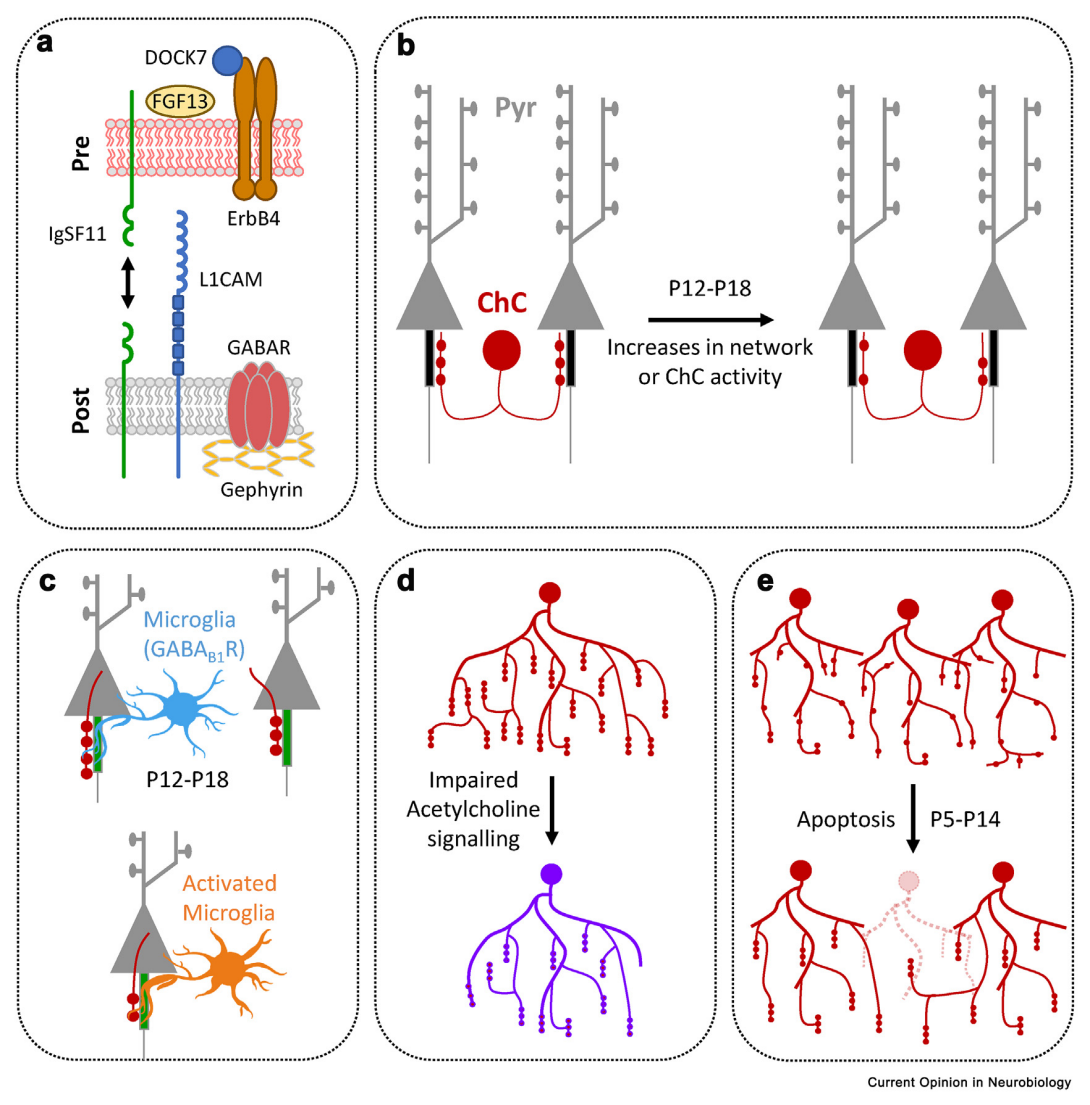
**Molecules and mechanisms regulating axo-axonic synapse formation along the AIS. a**. Molecules, expressed by ChCs (presynaptically) or by Pyramidal neurons (postsynaptically), involved in the proper establishment of axo-axonic synapses. **b**. Changes in both Pyr or ChC activity between P12 and P18 affect axo-axonic synapse formation. **c**. Presence of microglia along the AIS of Pyramidal neurons during development (P12–P18) favours the formation of axo-axonic synapses. Activation of microglia by LPS-induced inflammation leads to a pruning of these synapses. **d**. Acetylcholine signalling is necessary for the proper arborisation of ChC axons. **e**. In the binocular zone of the visual cortex, apoptosis of ChCs occurs during the second postnatal weeks, a time that coincides with axon arborisation and synapse formation.

Finding the molecules that allow interneurons to target the subcellular compartments of pyramidal cells with such specificity is an important question that remains an area of intense study [46]. Multiple approaches have been taken, each uncovering new molecules that play some role in the formation and/or stability of axo-axonic synapses (Figure 3a). Initial studies showed that Erbb4, a receptor tyrosine kinase expressed by PV interneurons, was important for the formation of axo-axonic synapses along the AIS [47,48]. Removal of Erbb4 from ChCs decreased the number of axo-axonic boutons but had no effect on axon arborisation or the number of pyramidal neurons contacted [47]. These findings were later extended to include DOCK7, a cytoplasmic driver of Erbb4 activity, shown to promote the formation of axo-axonic synapses [49]. A more recent study followed the developmental gene expression programs of three interneuron subtypes that target-specific subcellular compartments on principal neurons, to uncover genes upregulated during synaptogenesis [50•]. This study found mostly non-overlapping molecular programmes control the number of synapses formed by each interneuron subtype. In particular, FGF13 was shown to play an important role in controlling axo-axonic synapse number at the AIS, but not at other compartments [50•]. Using a similar gene-expression approach, but focusing exclusively on Cell Adhesion Molecules (CAMs), the homophilic adhesion protein IgSF11 was found to play a role in the proper arborisation of ChC axons and in axo-axonic synapse formation. Loss of IgSF11 resulted in a loss of axo-axonic synapses when removed from either ChCs or pyramidal neurons, and an enlargement of axo-axonic boutons when overexpressed specifically in ChCs [51•]. Interestingly, expression IgSF11 in deeper, non-target layers induced ectopic ChC synapses in these deeper layers, many of which targeted the soma, suggesting some role of this CAM in target specificity [51•]. A parallel approach carried out an *in vivo* RNAi screen of CAMs in pyramidal neurons to look for candidates that specifically affected axo-axonic synapse number [37•]. This study revealed the L1 family member, L1CAM, as a key molecule required for both the establishment and maintenance of axo-axonic synapses, through its anchoring to the AIS by the cytoskeletal ankyrin-G/bIV-spectrin complex. However, over-expression of L1CAM did not cause an increase in axo-axonic synapses, suggesting it may not be sufficient to drive the formation of synapses directly [37•]. From a synapse specificity point of view, many of these molecules are surprising candidates. They are typically not localised specifically at the AIS in pyramidal neurons, nor are they expressed exclusively in ChCs, and most have been implicated in the formation and plasticity of other synapse types [46]. Whilst these are all vital additions to our knowledge of the molecular repertoire that controls this synaptic compartment, the hunt for the mechanisms that can code for the localisation of synapses to specific subcellular compartments continues.

## Plasticity of axo-axonic synapse during development

Although the plasticity of excitatory synapses has been studied in great detail, our knowledge of the plasticity of GABAergic synapses is generally less well understood, particularly the axo-axonic synapses formed by ChCs. However, recent studies have shown that ChC axons are indeed sensitive to modulation by their local environment, particularly during development. For example, increases in cortical network activity, achieved by chemogenetic means, resulted in a loss of axo-axonic synapse number during synaptogenesis (P12–P18) (Figure 3b) [21•]. Since GABA is thought to be depolarising at the AIS during this period [20], the removal of synapses suggested that ChCs were likely following homeostatic plasticity rules. In fact, the same manipulation in activity in young adults (P40–P46), when GABA at the AIS is inhibitory, resulted in an increase in synapse number [21•]. In short, the polarity of this synapse dictated the direction of its plasticity and suggested that ChCs alter their output to stabilise the activity of pyramidal neurons and circuits [52]. Although the mechanisms behind this activity-dependent form of plasticity are not yet known, modulation of the levels of expression of any of the molecules described above could play a role. Another intriguing possibility would involve the participation of microglia, cells that have been shown to interact with the AIS [53] and have recently also been implicated in the formation and remodelling of GABAergic synapses [54, 55•, 56]. Indeed, microglia were shown to be needed for the proper formation and maintenance of synapses at the AIS, similar to findings in axo-dendritic synapses [56], and acute activation of these cells by lipopolysaccharide (LPS)-induced neuroinflammation decreased synapse number (Figure 3c) [55•]. Although no clear link yet exists between activity, microglia and axo-axonic synapse remodelling, it may be the case that hyperactivity of cortical networks results in the activation of microglia and the subsequent pruning of axo-axonic synapses during development, as seen at other synapses [57]. Alternative mechanisms, that are independent of neuronal activity, have also been uncovered that can modulate the axonal arborisation of ChCs early in development. For example, acetylcholine, a neurotransmitter with complex neuromodulatory effects in the cortex [58], was shown to promote the emergence of new filopodia during axon growth, thereby strongly modulating the extent of axonal arborisation of ChCs (Figure 3d) [59]. Although this phenotype did not require neuronal activity *per se*, it did rely on the local activation of axonal nicotinic acetylcholine receptors and calcium influx through T-type channels.

At a cell-wide level, activity also plays a role in establishing the overall number of ChCs found in the brain. Many interneurons in the cortex undergo apoptosis during a narrow window in development (around P5–P10) [60]. This developmental apoptosis can be modulated by network activity and serves to fine-tune the number of interneurons in the brain [61, 62, 63]. In most areas of the visual cortex, ChCs were shown to undergo developmental apoptosis during a similar period but, in the binocular zone of the visual cortex, apoptosis continued beyond P10, resulting in a further halving of ChC numbers by P14 (Figure 3e) [35•]. This protracted apoptosis was modulated by activity, such that retinal and callosal activities before eye opening drove apoptosis, and silencing these inputs kept more ChCs alive. It appears that the activity-dependent mechanisms at play are distinct from those observed in other interneurons during the earlier developmental apoptosis period, where increases in activity keep interneurons alive, providing a homeostatic control of the numbers of inhibitory interneurons in the brain [64,65]. The use of different rules in this later form of apoptosis is intriguing but perhaps not surprising, considering that it may well be playing a very different functional role. In this case, block of ChC apoptosis during this period caused deficiencies in the segregation of axonal projections from each eye and loss of binocularity [35•]. Understanding if this delayed apoptosis happens in other brain areas and how this cell death may impact ChC wiring more broadly will be important questions to tackle in the future.

## Outlook

ChCs have emerged from relative obscurity to provide a unique system for studying how interneurons wire up during development. Although the molecules involved and timing of these events are likely to be specific for different interneuron types, ChCs have the potential to give us a glimpse of how a specific interneuron type in the brain develops to form contacts with its target neurons. Their highly arborised and densely packed axon results in a high connection probability with neighbouring pyramidal cells, which facilitates the study of pre- and post-synaptic interactions, ideal for assessing the emergence of synapses. In addition, since contacts are formed exclusively at the AIS, the location of where synapses will emerge is well sign-posted and neatly compartmentalised. Finally, the fact that synapses form late in development and during a short temporal window, should help with the *in vivo* imaging of synapses, as they form. ChCs therefore have a number of unique features that can be exploited to better understand their wiring rules. Although still at an early stage, these emerging rules for axo-axonic synapses may help inform those of other interneurons in the brain.

## Conflict of interest statement

Nothing declared.

## Data Availability

No data was used for the research described in the article.
